# Supplementary material for: HDX-MS reveals nucleotide-dependent, anti-correlated opening and closure of SecA and SecY channels of the bacterial translocon
Source: eLife. 2019 Jul 10;8:e47402. doi: 10.7554/eLife.47402 (PMC6639072; doi:10.7554/eLife.47402)
Supplement: Supplementary file 1. — (A) Data is presented in Figure 2b. (B) HDX-MS data summary table for ΔHDX = (SecAAMPPNP +SecYEG) – (SecAADP +SecYEG). Data is presented in Figure 2c. Mass spectrometry raw data are deposited in the ProteomeXchange Consortium via the PRIDE database. [file elife-47402-supp1.pdf]

Supplementary file 1A. HDX data summary table for ΔHDX = (SecA^{AMPPNP}) – (SecA^{ADP}). Data is presented in **Figure 2b.** Mass spectrometry raw data are deposited in the ProteomeXchange Consortium via the PRIDE database.

ΔHDX = (SecAAMPPNP) – (SecAADP)		
	**SecAAMPPNP**	**SecAADP**
**HDX reaction details**	20 mM Tris pH 8, 2 mM MgCl2, 50 mM KCl, and 0.02% DDM	
**HDX time course (min)**	0.25, 1, 5 and 30 minutes	
**Back-exchange (mean/IQR)**	ND	
**Number of peptides**	215	215
**Sequence coverage**	81.8	81.8
**Average peptide length / Redundancy**	2.7	2.7
**Replicates (biological or technical)**	3 (technical)	3 (technical)
**Repeatability (average SD)**	0.072	0.07
**Significant differences in 30 min ΔHDX****	CI 99% = 0± 44 Da	

** To compare significant differences, a T-test with α=0.01 was used. Only peptides which satisfied a ΔHDX confidence interval of 99 % were considered significant.

Supplementary file 1B. HDX data summary table for ΔHDX = (SecA^{AMPPNP}+ SecYEG) – (SecA^{ADP}+SecYEG). Data is presented in **Figure 2c.** Mass spectrometry raw data are deposited in the ProteomeXchange Consortium via the PRIDE database.

ΔHDX = (SecA^{AMPPNP}+ SecYEG) – (SecA^{ADP}+SecYEG) *		
	**SecA^{AMPPNP} (+ SecYEG)**	**SecA^{ADP} (+SecYEG)**
**HDX reaction details**	20 mM Tris pH 8, 2 mM MgCl2, 50 mM KCl, and 0.02% DDM	
**HDX time course (min)**	0.25, 1, 5 and 30 minutes	
**Back-exchange (mean/IQR)**	ND	
**Number of peptides**	215	215
**Sequence coverage**	81.8	81.8
**Average peptide length / Redundancy**	2.7	2.7
**Replicates (biological or technical)**	3 (technical)	3 (technical)
**Repeatability (average SD)**	0.064	0.072
**Significant differences in 30 min ΔHDX****	CI 99% = 0± 41 Da	

* SecYEG were in excess (mixture of bond and unbound, therefore data not used).
** To compare significant differences, a T-test with α=0.01 was used. Only peptides which satisfied a ΔHDX confidence interval of 99 % were considered significant.
