# Supplementary material for: HDX-MS reveals nucleotide-dependent, anti-correlated opening and closure of SecA and SecY channels of the bacterial translocon
Source: eLife. 2019 Jul 10;8:e47402. doi: 10.7554/eLife.47402 (PMC6639072; doi:10.7554/eLife.47402)
Supplement: Supplementary file 2. — Data is presented in Figure 3. Mass spectrometry raw data are deposited in the ProteomeXchange Consortium via the PRIDE database. [file elife-47402-supp2.pdf]

Supplementary file 2. HDX data summary table for ΔHDX = (SecYEG + $SecA^{AMPPNP}$) – (SecYEG + $SecA^{ADP}$). Data is presented in **Figure 3.** Mass spectrometry raw data are deposited in the ProteomeXchange Consortium via the PRIDE database.

**ΔHDX = (SecYEG + $SecA^{AMPPNP}$) – (SecYEG + $SecA^{ADP}$)***		
	**SecY (+ $SecA^{AMPPNP}$)**	**SecY (+ $SecA^{ADP}$)**
**HDX reaction details**	20 mM Tris pH 8, 2 mM MgCl2, 50 mM KCl, and 0.02% DDM	
**HDX time course (min)**	0.25, 1, 5 and 30 minutes	
**Back-exchange (mean/IQR)**	ND	
**Number of peptides**	137	137
**Sequence coverage**	91.6	91.6
**Average peptide length / Redundancy**	2.91	2.91
**Replicates (biological or technical)**	3 (technical)	3 (technical)
**Repeatability (average SD)**	0.099	0.093
**Significant differences in 30 min ΔHDX****	CI 99% = 0± 62 Da	
	**SecE (+ $SecA^{AMPPNP}$)**	**SecE (+ $SecA^{ADP}$)**
**HDX reaction details**	20 mM Tris pH 8, 2 mM MgCl2, 50 mM KCl, and 0.02% DDM	
**HDX time course (min)**	0.25, 1, 5 and 30 minutes	
**Back-exchange (mean/IQR)**	ND	
**Number of peptides**	31	31
**Sequence coverage**	79.4	79.4
**Average peptide length / Redundancy**	2.34	2.34
**Replicates (biological or technical)**	3 (technical)	3 (technical)
**Repeatability (average SD)**	0.099	0.079
**Significant differences in 30 min ΔHDX****	CI 99% = 0± 63 Da	
	**SecG (+ $SecA^{AMPPNP}$)**	**SecG (+ $SecA^{ADP}$)**
**HDX reaction details**	20 mM Tris pH 8, 2 mM MgCl2, 50 mM KCl, and 0.02% DDM	
**HDX time course (min)**	0.25, 1, 5 and 30 minutes	
**Back-exchange (mean/IQR)**	ND	
**Number of peptides**	25	25
**Sequence coverage**	95.5	95.5
**Average peptide length / Redundancy**	2.11	2.11
**Replicates (biological or technical)**	3 (technical)	3 (technical)
**Repeatability (average SD)**	0.101	0.112
**Significant differences in 30 min ΔHDX****	CI 99% = 0± 64 Da	

* SecA is in excess (mixture of bond and unbound, therefore data not used).
** To compare significant differences, a T-test with α=0.01 was used. Only peptides which satisfied a ΔHDX confidence interval of 99 % were considered significant
